# A New Form of Suture

**Published:** 1867

**Authors:** Albert H. Hoy

**Affiliations:** Racine, Wisconsin


*A New Form of Suture.* By ALBERT H. HOY, M.D., of Racine, Wisconsin.

While serving on the Medical Staff of the U.S. Army, during the late war, we repeatedly used a suture to which, not knowing of its being mentioned in any treatise on surgery, we desire to call the attention of the profession, trusting that they will find that it meets the indications of a good suture, *viz.:*—

1st. Adaptation of the edges of the wound.

2d. Producing little irritation.

3d. Easily applied.

We term it the *Rubber Suture*, and in using it, the following things are required:—

1st. A paper of ladies' sewing needles, the points of which, for one-third of their length, have been heated in a lamp and curved, like a surgeon's needle. No. 4 is the best size.

2d. Some pure rubber elastic bands, cut into inch lengths. That having a width of one-tenth of an inch will be found most generally useful.

3d. A pair of small pliers and wire-cutters.

The suture is introduced in the following manner:—One of the needles is taken firmly by the eye with the pliers, and a piece of the rubber band is strung on, near one of its ends; the needle, thus armed, is thrust through the edges of the wound, holding them together; the free end of the rubber band is then strung over the point of the needle, care being taken to give the band just sufficient tension to hold the lips of the wound snugly together; the points of the needles are then snipped off with the cutters, and the suture is complete.

In place of the rubber bands, what is known as the French rubber tubing, for dental purposes, is, in some cases, preferable. Sections of this, of size in accordance with the tension required, may be looped over the ends of the needles after they are introduced, by means of the artery forceps.

It may not be out of place, to remark that we have seen numerous instances of what might be properly termed union by the first intention, in cases where this suture was used, and these, too, in the most extensive wounds. The elasticity of the rubber tends constantly to keep the lips of the wound in direct apposition, even though the needles may be loosened by suppuration, and it is well known that steel or iron produces a very trifling amount of irritation. The rubber, also, is not affected by the heat or secretions of the part.

We were led, just now, to call attention to this suture, from the fact that a few days ago we assisted Dr. P. R. Hoy, of this city, in removing nearly the entire under lip of a gentleman, for an epithelial cancer, and succeeded, after loosening the integuments from the lower jaw, in bringing the lips of the wound together, and retaining them perfectly successfully until union took place, by means of this rubber suture. We desire to recommend it strongly in hare-lip and other plastic operations, where a speedy union of cut surfaces is of the greatest importance.

